# Postpartum Symphysis Pubis Diastasis: A Case Report

**DOI:** 10.31729/jnma.8375

**Published:** 2023-12-31

**Authors:** Babita Chaudhary, Sumit Bidari, Sujata Maharjan, Gauri Adhikari, Lokendra Bata

**Affiliations:** 1 Department of Obstetrics and Gynaecology, Nepalese Army Institute of Health Sciences, Sanobharyang, Kathmandu, Nepal; 2 Department of Obstetrics and Gynaecology, Kathmandu Medical College and Teaching Hospital, Sinamangal, Kathmandu, Nepal; 3 Department of Internal Medicine, Nidan Hospital Limited, Pulchowk, Lalitpur, Nepal

**Keywords:** *case reports*, *pelvic pain*, *pubic symphysis diastasis*

## Abstract

The pubic symphysis is a midline, non-synovial joint connecting the right and left superior pubic rami. The joint allows very limited movement of approximately 0.5-1 mm. Under hormonal stimulation during pregnancy, the widening of the symphysis pubis and sacroiliac joints occurs. Pubic symphysis diastasis is defined as the widening of the pubic joint of >10 mm. It is a rare complication of vaginal childbirth for which no gold standard treatment has been defined. Most cases are treated conservatively. A case of pubic diastasis in a 24-year-old G5P2A2L1 following vaginal delivery is reported. Management consisted of simple conservative treatment, which was sufficient in achieving symptomatic relief.

## INTRODUCTION

Pubic symphysis diastasis (PSD) following childbirth via vaginal delivery is a rare but debilitating condition. Widening of the cartilaginous joint during pregnancy before childbirth is physiologic and assists in expanding the birth canal for successful delivery.^1^ However, reports of non-physiologic pubic diastasis exceeding that typically required for childbirth (greater than 1 cm) can leave mothers with debility and extreme pain. The incidence of complete separation of the pubic symphysis is reported to be 1 in 300 to 1:30,000, with many instances likely undiagnosed.^1^

Discussions of multiple treatment options in the literature include non-operative treatment with application of pelvic binder coupled with physical therapy and immediate weight bearing, non-weight bearing with bedrest, closed reduction with application of binder, application of anterior external fixator with or without sacroiliac screw fixation, and anterior internal fixation with plate and screws.^2^

## CASE REPORT

**Figure 1:**
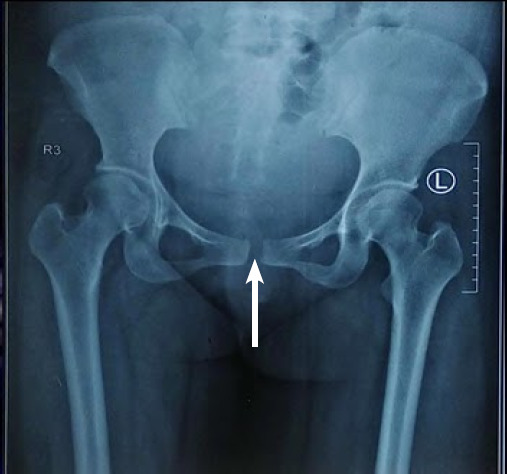
Anteroposterior radiograph of the pelvis showing pubic symphysis diastasis and sacroiliac joint shows no fracture or widening.

A 24-year-old G5P2A2L1 at 39+^4^ weeks with no comorbidities was admitted to the hospital with labour pain. Her labour progressed slowly and she delivered a 3.4 kg baby by normal vaginal delivery without any complications. Her first stage of labour lasted for 6 hours and her second stage about 20 minutes with an uneventful immediate postpartum period. The very next day she complained of pain in the bilateral hip region, more during movements with difficulty in walking and also passing urine. On examination she had severe tenderness over the pubic symphysis and difficult leg raising with restricted range of movements of the hip joint. Orthopedics opinion was sought and an x-ray pelvis anteroposterior view was taken. The x-ray demonstrated a wide gap of 1.6 cm between the pubic bones and the condition was diagnosed as symphysis pubis diastasis (Figure 1).

**Figure 2:**
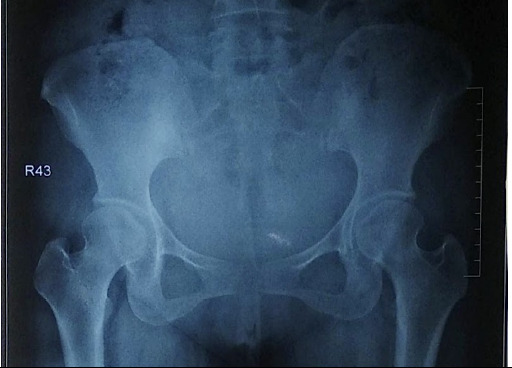
Anteroposterior radiograph of the pelvis at discharge.

She was treated symptomatically with bed rest, analgesics, anti-inflammatory and pelvic binders. She was discharged on day 20 when she felt symptomatically better. A repeat x-ray was taken before discharge (Figure 2).

She was reviewed after one month; she was able to walk without any support. Weight-bearing activity, climbing stairs, and squatting were still not possible. The patient was again followed up after one month when she was able to walk upstairs without support and was able to squat and perform weight-bearing activities as well.

## DISCUSSION

The pubic symphysis is a non-synovial joint that connects the right and left superior pubic rami with a normal radiographic separation width of 4 to 5 mm.

Due to hormone-related changes and physiological alterations observed during pregnancy, the gap can increase by 2-3 mm and remain so after delivery, such a separation is called physiological pubic symphysis diastasis. Infrequently, vaginal delivery might lead to joint widening of >10 mm which is diagnostic and defined as pathological pubic symphysis diastasis.^3^

According to a study that assessed the dynamics of pubic symphysis during labour and postpartum, using ultrasonography, widening of symphysis took place in 59 to 94% of parturients depending upon the exact site studied. The magnitude of the widening varied from 9 to 139% of the original gap.^4^ However, several studies have demonstrated that there is no definite correlation between the magnitude of separation and the severity of symptoms.^5,6^

Factors contributing to symphysis pubis diastasis are multiparity, cephalopelvic disproportion, precipitate labour, difficult labour, difficult forceps delivery and pre-existing pathology of pelvic bones.^7^ The most common factors contributing to the condition are hormonal and Mc Roberts maneuver, which is generally safe but may result in pubic symphysis diastasis when there is prolonged placement of the patient's legs in hyper flexed position.^8^

The usual presentation is that of something giving way in the region of the symphysis pubis sometimes with an audible crack at the time of delivery. Unbearable pain on moving from side to side and on performing any weight-bearing activity (such as walking or climbing stairs) precludes ambulation in the immediate postpartum period. This could be accompanied by disruption of the sacroiliac joint, haemorrhage, or urine incontinence in severe cases. Radiography, ultrasound, and magnetic resonance imaging are the diagnostic modalities that aid confirmation of diagnosis. The magnitude of separation does not correlate well with the severity of symptoms.

Treatment modalities range from conservative management (including analgesics, pelvic binders and transcutaneous nerve stimulation) and chiropractic management to orthopaedic interventions such as external fixation or open reduction and internal fixation. Since postpartum pain is frequently dismissed as attributable to labour and childbirth, the diagnosis of pubic symphysis diastasis is often delayed and sometimes missed altogether.^1^ Rarely non-traumatic pubic diastasis may require surgical correction with open reduction and internal fixation or wiring if the diastasis is greater than 2.5 cm not responding to conservative management after 6 weeks.^9^

Pubic symphysis diastasis is an uncommon injury that should be considered when evaluating patients in the peripartum period who are experiencing suprapubic, sacroiliac or thigh pain. This report validates that non-traumatic symphyseal rupture following vaginal delivery can be managed satisfactorily, without any operative intervention or prolonged bed rest. There is a need for awareness among medical professionals about the condition as this has increased the incidence of recurrence in subsequent pregnancies.

## References

[1] Chawla JJ, Arora D, Sandhu N, Jain M, Kumari A (2017). Pubic symphysis diastasis: a case series and literature review.. Oman Med J..

[2] Seidman AJ, Siccardi MA (2023). Postpartum Pubic Symphysis Diastasis..

[3] Jain S, Eedarapalli P, Jamjute P, Sawdy R (2011). Symphysis pubis dysfunction: a practical approach to management.. The Obstetrician and Gynaecologist..

[4] Rustamova S, Predanic M, Sumersille M, Cohen WR (2009). Changes in symphysis pubis width during labor.. J Perinat Med..

[5] Hagen R (1974). Pelvic girdle relaxation from an orthopaedic point of view.. Acta Orthop Scand..

[6] Scriven MW, Jones DA, McKnight L (1995). The importance of pubic pain following childbirth: a clinical and ultrasonographic study of diastasis of the pubic symphysis.. J R Soc Med..

[7] Jayaraman JK, Ganapathy P, Indira N (2015). Post-partum diastasis of the pubic symphysis: report of a rare case.. J Clin Diagn Res..

[8] Gupta P, Malik R (2019). Case series of pubic bone diastasis causing severe pelvic girdle pain in pregnancy.. Int J Reprod Contracept Obstet Gynecol..

[9] Heath T, Gherman RB (1999). Symphyseal separation, sacroiliac joint dislocation and transient lateral femoral cutaneous neuropathy associated with McRoberts' maneuver.. a case report. J Reprod Med..

